# Single screening versus conventional double screening for study selection in systematic reviews: a methodological systematic review

**DOI:** 10.1186/s12874-019-0782-0

**Published:** 2019-06-28

**Authors:** Siw Waffenschmidt, Marco Knelangen, Wiebke Sieben, Stefanie Bühn, Dawid Pieper

**Affiliations:** 1 Institute for Quality and Efficiency in Health Care, Cologne, Germany; 2 Institute for Research in Operative Medicine Witten/Herdecke University, Cologne, Germany

**Keywords:** Systematic reviews, Study selection, Methodology

## Abstract

**Background:**

Stringent requirements exist regarding the transparency of the study selection process and the reliability of results. A 2-step selection process is generally recommended; this is conducted by 2 reviewers independently of each other (conventional double-screening). However, the approach is resource intensive, which can be a problem, as systematic reviews generally need to be completed within a defined period with a limited budget. The aim of the following methodological systematic review was to analyse the evidence available on whether single screening is equivalent to double screening in the screening process conducted in systematic reviews.

**Methods:**

We searched Medline, PubMed and the Cochrane Methodology Register (last search 10/2018). We also used supplementary search techniques and sources (“similar articles” function in PubMed, conference abstracts and reference lists). We included all evaluations comparing single with double screening. Data were summarized in a structured, narrative way.

**Results:**

The 4 evaluations included investigated a total of 23 single screenings (12 sets for screening involving 9 reviewers). The median proportion of missed studies was 5% (range 0 to 58%). The median proportion of missed studies was 3% for the 6 experienced reviewers (range: 0 to 21%) and 13% for the 3 reviewers with less experience (range: 0 to 58%).

The impact of missing studies on the findings of meta-analyses had been reported in 2 evaluations for 7 single screenings including a total of 18,148 references. In 3 of these 7 single screenings – all conducted by the same reviewer (with less experience) – the findings would have changed substantially. The remaining 4 of these 7 screenings were conducted by experienced reviewers and the missing studies had no impact or a negligible on the findings of the meta-analyses.

**Conclusions:**

Single screening of the titles and abstracts of studies retrieved in bibliographic searches is not equivalent to double screening, as substantially more studies are missed. However, in our opinion such an approach could still represent an appropriate methodological shortcut in rapid reviews, as long as it is conducted by an experienced reviewer. Further research on single screening is required, for instance, regarding factors influencing the number of studies missed.

**Electronic supplementary material:**

The online version of this article (10.1186/s12874-019-0782-0) contains supplementary material, which is available to authorized users.

## Background

A systematic, reproducible and transparent methodological approach is a key component in systematic reviews. The systematic review process consists of several steps: after a systematic search for the relevant literature, the publications retrieved are screened and the relevant ones selected. This is followed by data extraction and analysis as well as an appraisal of the review’s results.

Stringent requirements exist with regard to the transparency of the study selection process (hereinafter referred to as “screening”) and the reliability of the corresponding results. These requirements aim to avoid the non-detection of relevant evidence with a subsequent risk of bias that endangers the validity of conclusions drawn from the evidence available [1, 2].

The relevant publications are selected in several steps [3]:

- Exclusion of irrelevant references (i.e. references not fulfilling the eligibility criteria) through perusal of the titles, and, if available, the abstracts. If doubts exist as to the relevance of a study, the corresponding full text is obtained.
- The full texts of the potentially relevant publications are obtained. The decision on the inclusion of the study is then made on the basis of these full texts.

All selection steps are performed by 2 persons independently of each other. Discrepancies are resolved by discussion.

The double screening approach is an international standard and recommended by well-established handbooks, which mostly refer only to the study by Edwards 2002 as the evidence base for this recommendation [4].

The double-screening approach offers the following advantages: firstly, it ensures that the study inclusion criteria are applied consistently, thus avoiding systematic errors, and secondly, random errors such as careless mistakes can be identified and corrected [5]. However, it is resource intensive, which can be a problem, as systematic reviews generally need to be completed within a defined period with a limited budget [1, 2].

In recent years, the focus of methodological research has shifted more to the analysis of efficiency resources, as there is a growing need to provide evidence products faster [6–9], for instance, as rapid reviews. This means that there is an increasing demand for research on methodological shortcuts. Its aim is to evaluate what impact these shortcuts have on the validity of the results and conclusions of systematic reviews [6, 8, 9]. Single screening, which requires far fewer resources than double screening, also represents a potential shortcut [7, 10, 11]. It is therefore of interest whether, and under what conditions and with what impact, a single screening approach could be applied.

The aim of the following methodological systematic review was to analyse the evidence available on whether single screening is equivalent to double screening in the screening process conducted in systematic reviews.

## Methods

### Information sources and literature search

The electronic search strategy was developed by an experienced information specialist (SW). We searched Medline (Ovid), all PubMed databases, and the Cochrane Methodology Register (see Additional file 1: Appendix A); the last bibliographic search was conducted in October 2018. We also applied the “similar articles” function in PubMed with 4 known key publications to identify additional relevant articles (applied for the first 20 entries). Furthermore, in June 2018 we searched all Cochrane Colloquium abstracts (since 2009) as well as the Cochrane database of oral, poster and workshop presentations (since 1994). We also cross-checked reference lists of all articles included. In addition, we screened guidelines known to us on the conduct of systematic reviews.

### Eligibility criteria and selection of evaluations

We included all evaluations comparing single with double screening (i.e. including at least 2 reviewers screening independently of one another). We did not limit the evaluations to a certain number of screening steps, i.e. evaluations dealing only with one screening step (e.g. title/abstract screening) were eligible for inclusion. Evaluations involving students or persons without screening experience were excluded.. The rationale behind this decision was that we were only interested in testing standards for a highly professional environment (e.g. an HTA agency) and not in whether or how inexperienced researchers could be involved in the screening process. No text mining or automation tools were allowed. No restrictions to the type of studies to be screened in the evaluations (e.g. therapeutic) were applied. Only evaluations published in English and German were included.

As a minimum requirement, each evaluation had to report at least one quantitative measure for missing studies. No data on the agreement between reviewers (e.g. calculation of Cohen’s kappa) were considered, as they were not the focus of our study.

We expected the most frequent comparison to be single versus double screening (i.e. the gold standard applied in the evaluations; see Table 1 for definitions). Additional analyses could include an assessment of the impact of the non-detection of relevant studies, for example, by investigating whether this would have led to changes in the results of a meta-analysis of the study pool originally included. Additional file 2: Appendix B outlines the eligibility criteria in detail.

**Table 1:** Characteristics of the 4 evaluations

	Edwards 2002	Doust 2005	Pham 2016	Shemilt 2016
Aim of evaluation	Estimate the accuracy and reliability of reviewers when screening records for relevant trials for a systematic review	Assess the reliability and accuracy of reviewers’ screening	Assess the implications of applying methodological shortcuts: one of the shortcuts is a single screening approach	Compare the costs and effects of a single screening approach.
Other aims/comparisons included in the evaluation	–	The other aim was to assess the sensitivity and precision of five published search strategies	The other shortcuts were: - one bibliographic database plus ancillary sources - limiting the search to bibliographic databases - only papers available electronically	Four variant screening approaches were analysed. The other approaches were: - safety first screening - double screening - single screening with text mining
Number of - reviews examined	- 1	- 2	- 3	- 1
- reviewers involved	- 4	- 2	- 2	- 1
- sets of screenings examined	- 6*	- 2	- 3	- 1
- individual screenings analysed	- 12	- 4	- 6	- 1
Screening step assessed	Title/abstract screening only	Title/abstract screening only	Title/abstract screening only	Title/abstract screening only
Piloting screening	Prior meeting to discuss inclusion criteria	No information provided	Pre-test screening of 50 potentially relevant records	No information provided
Gold standard	Studies identified as relevant by a double screening approach.Disgreements were resolved by consensus	Studies identified as relevant by a double screening approach. Disagreements were resolved by consensus.	Original reviews’ study pool. No further information provided.	Studies identified as relevant by a double screening approach. Disagreements were resolved by consensus.
Number of hits needed to be screened	22,571 hits, (each reviewer was to screen approximately 11,286 records)	Tympanometry: 638 hits Natriuretic peptides: 373 hits	Wilhelm 2011: 1890 hits Greig 2012: 3091 hits Bucher 2015: 690 hits	12,477 hits
Study type(s) included in the review	RCTs	Diagnostic test accuracy studies	Wilhelm 2011: n/a; no limitations Greig 2012: limited to experimental (control and challenge trials), quasi-experimental (before-and-after trials), cohort designs Bucher 2015: no limitations on study designs	Studies of any design, using quantitative and/or qualitative methods
Reviewer experience as reported in the evaluation	“Each reviewer had substantial experience of screening records for systematic reviews, apart from reviewer 2 who was relatively inexperienced.”	“The first reviewer had more content knowledge and more experience in completing systematic reviews” [...]	“Reviewer A was a veterinarian, had a master’s degree in epidemiology and had over 5 years of experience in relevance screening for reviews in agri-food public health. Reviewer B had a master’s degree in public health and over 2 years of experience in relevance screening for reviews in agri-food public health”.	[…] “conducted by an experienced team of systematic reviewers with substantial experience in primary care and medical education”
Reviewer experience: classification	Reviewer 1: experienced Reviewer 2: less experienced Reviewer 3: experienced Reviewer 4: experienced	Reviewer 1: experienced Reviewer 2: less experienced	Reviewer 1: experienced Reviewer 2: less experienced	Reviewer 1: experienced
Re-analysis meta-analysis	No	No	Yes	Yes
Number of missed records presented	Yes	Yes	Yes	Yes

*Each reviewer was assigned to 3 sets to screen (2 reviewers for each set): Reviewer 1: sets A,B,C; Reviewer 2: sets A,D,E; Reviewer 3: sets B, D, F; Reviewer 4: sets C, E, F

We used an online screening tool for the screening process (an internal tool called web Trial Selection Database, webTSDB). All titles/abstracts identified in the electronic databases were screened by 2 authors (DP, SB) independently of one another. Discrepancies were resolved by discussion. Cochrane Colloquium abstracts and the database of oral poster and workshop presentations were screened by one author (SB), which is in line with recommendations for the screening of supplementary information sources [3]. All potentially relevant full texts were screened by 2 authors (DP, SB) independently of one another. Discrepancies were resolved by discussion. In the case of discrepant judgements, a third author (SW) was involved.

### Data extraction and synthesis

Data extraction was conducted by one author (SW) and checked by another (DP). Data were summarized in a structured narrative way. The narrative synthesis included information on the sample (evaluations, sets for screening, and studies included), reviewers, screening methods, the gold standard as well as results. In addition, we performed a post-hoc subgroup analysis to investigate the impact of reviewer experience. No assessment of risk of bias or methodological quality was performed due to the nature of our review and the wide range of study designs included in the evaluations analysed.

We calculated the median proportion of missed studies with respect to all screenings. As there were great variations in the number of missed studies between reviewers, we conducted post-hoc subgroup analyses based on reviewer experience.

We did not register our review in the International Prospective Register of Systematic Reviews (PROSPERO), as it did not meet the eligibility criteria (inclusion of at least one outcome of direct patient or clinical relevance). The current systematic review was conducted in accordance with the Preferred Reporting Items for Systematic Reviews and Meta-Analyses (PRISMA) statement (Additional file 3: Appendix C).

## Results

**Fig. 1:**
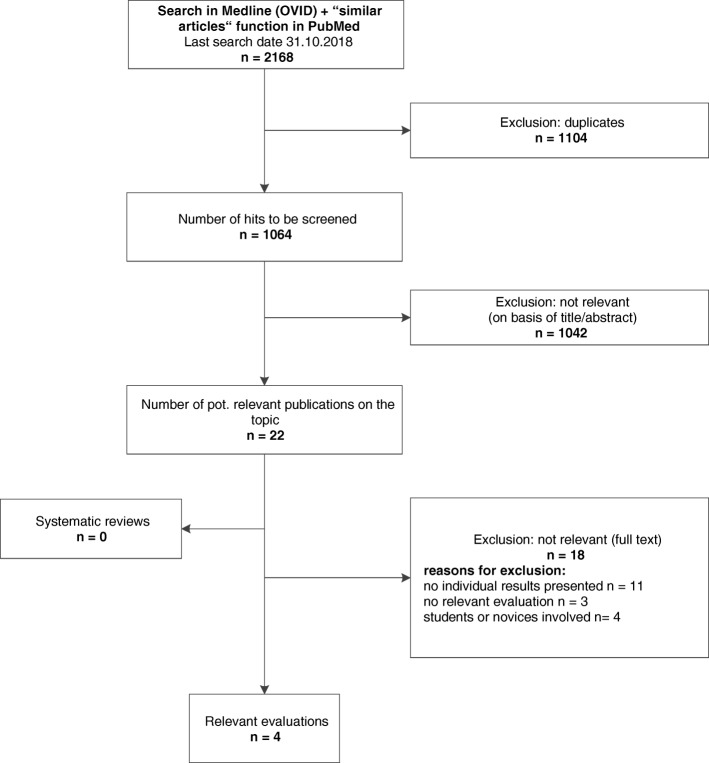
Flowchart for selection of evaluations of screening approaches

The bibliographic search yielded 2168 hits; 1064 hits without duplicates were screened; 22 were potentially relevant and obtained in full text (Fig. 1). Subsequent full paper-based screening excluded an additional 18 references, as no relevant evaluation was reported (*n* = 3) or potentially relevant evaluations involved students or persons without screening experience (*n* = 4) or quantified measures were either not reported or could not be calculated with the results reported (*n* = 11); see Additional file 4: Appendix D. We identified one potentially eligible evaluation reported in a conference abstract, with no full-text publication available. We contacted one of the authors who responded that he had no access to any data. No additional evaluations were identified through handsearching or any other sources.

We ultimately included 4 evaluations (Edwards 2002 [12], Doust 2005 [13], Pham 2016 [11], Shemilt 2016 [10]).

The characteristics of the 4 evaluations included are presented in Table 1. The 4 evaluations investigated a total of 23 single screenings (12 sets for screening conducted by 9 reviewers). The number of hits needed to be screened varied between 373 and 12,477 hits for each reviewer per set. All evaluations only examined title and abstract screening.

The 4 evaluations considered different study types: randomized controlled trials (RCTs) (Edwards 2002), diagnostic test accuracy studies (Doust 2005), as well as all study types (Pham 2016 and Shemilt 2016). No patterns or associations were noticeable between study types screened in the evaluations and the number of studies missed.

The reviewers’ level of experience in the 4 evaluations varied. Six of the 9 reviewers were described as being experienced and 3 had a lower level of experience than the other reviewer(s) involved (see Table 1). All evaluations reported the number of studies missed by the reviewers. Two re-ran the meta-analysis without the missed studies (Pham 2016, Shemilt 2016).

Table 2 summarizes the overall results and the results of the individual evaluations. Edwards 2002 is the only evaluation with a comparable research question and their results are similar to ours. That was to be expected, as Edwards 2002 contributed more than half (12 of 23) of the individual screenings to our overall result.

**Table 2:** Median proportion of missed studies

	Median proportion missed	Sets of screenings	Min. in %	Max. in %
Overall result	5%	23	0%	57.8%
Edwards 2002	5.7%	12	0%	24.1%
Doust 2005	1.5%	4	0%	3.0%
Pham 2016	16.6%	6	0%	57.8%
Shemilt 2016	0.6%	1	**–**	**–**

The number of missed studies in each set for screening is displayed in Table 3 (summarized in Table 2). In 23 screenings conducted by 9 reviewers, 41,730 references were screened; the median proportion of missed studies was 5% (range 0 to 58%).

**Table 3:** Individual results of the evaluations

Evaluation	Reviewer	Review or set of screenings	Number of missed studies	Number of included studies (gold standard)	Proportion of missing studies	Results of the re-analysis of the meta-analysiszz
Edwards 2002	1	Set A	0	22	0%	n.a.
	1	Set B	1	30	3%	n.a.
	1	Set C	3	31	10%	n.a.
	2**	Set A	2	22	9%	n.a.
	2**	Set D	2	20	10%	n.a.
	2**	Set E	7	29	24%	n.a.
	3	Set B	1	30	3%	n.a.
	3	Set D	1	20	5%	n.a.
	3	Set F	5	24	21%	n.a.
	4	Set C	2	31	6%	n.a.
	4	Set E	0	29	0%	n.a.
	4	Set F	1	24	4%	n.a.
Doust 2005	1	Tympanometry	1	33	3%	n.a.
	2**	Tympanometry	1	33	3%	n.a.
	1	Natriuretic peptides	0	20	0%	n.a.
	2**	Natriuretic peptides	0	20	0%	n.a.
Pham 2016	1	Wilhelm 2011	2	19	11%	Negligible impact on findings
	1	Greig 2012	2	36	6%	Negligible impact on findings
	1	Bucher 2015	0	18	0%	No impact on findings
	2**	Wilhelm 2011	11	19	58%	Substantial change in findings
	2**	Greig 2012	7	36	19%	Substantial change in findings
	2**	Bucher 2015	3	18	17%	Substantial change in findings
Shemilt 2016	1	Park 2015	1	169	1%	Negligible impact on findings
Overall Result			**53**	**733**		

** Reviewer with less experience than the other reviewer(s) involved

n.a. not applicable

The post-hoc subgroup analyses based on reviewer experience showed that 15 of the 23 screenings were conducted by 6 experienced reviewers and 8 by the 3 reviewers with less experience. The median proportion of missed studies for the experienced reviewers was 3% (range: 0 to 21%) and 13% for the 3 other reviewers (range: 0 to 58%).

The impact of missing studies on the findings of meta-analyses had been reported in 2 evaluations for 7 single screenings including a total of 18,148 references. In 3 of these 7 single screenings – all conducted by the same reviewer (with less experience) – the findings would have changed substantially. The remaining 4 of these 7 screenings were conducted by experienced reviewers and the missing studies had no impact or a negligible one on the findings of the meta-analyses.

## Discussion

Our methodological systematic review of evaluations of single versus double screening showed that single screening of the titles and abstracts of studies retrieved in bibliographic searches is not equivalent to double screening, as substantially more studies are missed. However, our findings indicate that this approach could still represent a potential approach for study selection, as long as it is conducted by an experienced reviewer.

### Reviewer experience

Only 2 of the 4 evaluations included re-analysed data without the missing studies. In 3 of the 7 single screenings, the studies missed would have led to a substantial change in the findings of the meta-analyses. Even though the reviewer responsible was less experienced than the other reviewer involved, the number of studies missed was surprising. For example, he or she missed 11 of 19 studies in the Wilhelm 2011 review ([14] in Pham 2016). In comparison with the results of the other evaluations, this is a major outlier (the results for the other inexperienced reviewer ranged from 3 to 24% missed studies).

Pham did not provide an explanation for this, but even if study inclusion criteria had been applied inconsistently or random errors had occurred, this high number of studies missed is unusual. As this evaluation was the only one with pre-test screening, any topic-related systematic error should have been eliminated.

It has also been shown that reviewer experience has an impact on other tasks in systematic reviews, such as assessing their methodological quality or extracting data [15].

### Research question too vague

One explanation why studies were missed might be that the research question was too vague and largely depended on the reviewer’s interpretation. Pham stated “the specificity of the review question may have made identification of relevant studies more straightforward for reviewers” [11]. This is supported by the fact that the Bucher 2015 review, which yielded the best results for single screening, had the most narrowly defined research question of all 3 reviews included by Pham, with just one population group, one intervention and one pathogen. Patients, interventions, comparisons and outcomes (PICO) should therefore be defined as exactly as possible in order to avoid dependence on the reviewer’s interpretation of exactly what study types, interventions or, in this example, pathogens might be eligible.

### Single screening approach as a methodological shortcut

The question remains as to whether it is an appropriate decision to apply single screening of titles and abstracts as a methodological shortcut for rapid reviews. As Shemilt 2016 concluded, such a decision depends on “the willingness of review teams and funders to sacrifice recall in order to substantively reduce the overall workload and total costs of systematic review production”. In our opinion, the reduction in recall is marginal and the results are robust enough to establish this approach as a methodological shortcut, as long as it is applied by an experienced reviewer.

### Importance of bibliographic searches

A further aspect should also be considered in future research: all results of the 4 evaluations included refer to the screening of citations retrieved from bibliographic databases as the only information source. However, systematic reviews generally consider several other sources (e.g. clinical study reports provided by regulatory agencies or manufacturers, study registries, scanning reference lists etc.), so that the identification of the relevant study pool does not rely solely on the screening approach for the results of the bibliographic search. The impact of these additional searches on the number of missing studies is not mentioned in the evaluations analysed. However, there is evidence that these different search approaches (e.g. citation searching) could represent useful supplementary alternatives [16]. It should also be noted that in the assessment of drugs, bibliographic databases provide insufficient information to enable the assessment of a primary study and should therefore not be the main information source [17]. None of the 4 evaluations we included mentions this aspect, even though other information sources had also been considered (e.g. Pham 2016 evaluated the screening in Greig 2012, in which reference lists and conference proceedings had also been screened). The impact of studies missed in the screening of results of bibliographic searches may thus be lower than expected when other information sources, which may contain the missing studies, are considered.

### Available evidence

We could compare our results only with one other systematic review. Recently, Robson 2018 summarized evaluations of methods for systematic reviews, including study selection. According to their results on screening, the evidence supported the involvement of 2 independent experienced reviewers. Robson 2018 included 4 studies to investigate the question as to whether 2 independent reviewers are required for study selection. They included Yip 2013 [25], which we excluded due to the lack of a quantitative measure for missing studies. In addition, we included one further evaluation (Pham 2016) not included in Robson. Robson summarized the conclusions of the evaluations included, whereas we extracted and analysed the actual data. Our findings may thus potentially provide a more accurate picture of the current evidence. However, we emphasize that our findings can only indicate certain tendencies or be used to help create hypotheses for future research to test when a single screening approach might be applicable.

### Research gaps

Evidence is still lacking on the issue as to whether the number of missed studies would change if full-text screening were also performed by a single reviewer.

A further important issue is the technical aspect of screening. Except for Shemilt 2016, none of the evaluations reported whether they had used a screening tool, reference management software, or hardcopies for screening. It can be assumed that 15 to 20 years ago (applies to Edwards 2002 and Doust 2005) screening was conducted using hardcopies, an approach that might be more error prone than the use of a screening tool. Edwards 2002 noted that aspects of electronic records could influence their ease of identification for systematic reviews.

#### Future research

There is still a need for further validation of the single screening approach under consideration of the following factors:

- the impact of reviewer experience and poorly described PICO on the number of missed studies,
- the impact of missed studies on results of meta-analyses,
- the impact of non-bibliographic information sources on the relevance of studies missed in bibliographic searches,
- the impact of single full-text screening on sensitivity (vs. double screening)
- the impact of training or piloting before starting screening
- the impact of screening tools
- the impact of the prioritization of references in combination with single or double screening (as analysed in Shemilt 2016).

We are therefore currently conducting further research on screening approaches, including single screening, to address these open questions [18].

### Limitations

Our work has some limitations: firstly, searching for evaluations on screening approaches is challenging. We tried to identify all relevant sources; however, we cannot exclude that we missed some relevant evaluations. Secondly, we had to rely on the information provided in the evaluations included; re-analyses were not possible due to the way results were reported. Thirdly, we could only roughly classify reviewer experience, as the information provided in the evaluations was inconsistent and incomplete: for instance, only one evaluation reported the extent of screening experience in years and none reported the number of systematic reviews previously conducted.

## Conclusions

Single screening of the titles and abstracts of studies retrieved in bibliographic searches is not equivalent to double screening, as substantially more studies are missed. However, in our opinion such an approach could still represent an appropriate methodological shortcut in rapid reviews, as long as it is conducted by an experienced reviewer. The current evidence base on the impact of studies missed in screening is insufficient and further research is required to confirm our preliminary findings. There is also a need for further validation of the single screening approach, for example, by investigating factors that influence the number of studies missed in screening.

## Additional files

Additional files 1: Appendix A: Search strategy (DOCX 22 kb)

Additional files 2: Appendix B: Detailed eligibility criteria (DOCX 14 kb)

Additional files 3: Appendix C: Completed PRISMA 2009 Checklist (DOC 67 kb)

Additional files 4: Appendix D: List of excluded references (full text) sorted by reasons (DOCX 21 kb)

## Data Availability

All data generated or analysed during this systematic review are included in this published article.

## Abbreviations

PICO: patient, intervention, comparison and outcome
RCT: randomized controlled trials

## References

[1] O'Mara-Eves A, Thomas J, McNaught J, Miwa M, Ananiadou S (2015). Using text mining for study identification in systematic reviews: a systematic review of current approaches. Syst Rev..

[2] Olofsson H, Brolund A, Hellberg C, Silverstein R, Stenström K, Österberg M (2017). Can abstract screening workload be reduced using text mining? User experiences of the tool Rayyan. Res Syn Meth.

[3] Institut für Qualität und Wirtschaftlichkeit im Gesundheitswesen. General methods: version 5.0. 2018; https://www.iqwig.de/download/General-Methods_Version-5-0.pdf. Accessed 06.06.2018.

[4] Edwards P, Clarke M, DiGuiseppi C, Pratap S, Roberts I, Wentz R (2002). Identification of randomized controlled trials in systematic reviews: accuracy and reliability of screening records. Stat Med.

[5] McDonagh M, Peterson K, Raina P, Chang S, Shekelle P. Avoiding bias in selecting studies. Rockville, MD: Agency for Healthcare Research and Quality; 2013. https://effectivehealthcare.ahrq.gov/sites/default/files/pdf/methods-guidance-bias_methods.pdf. 23487864

[6] Garritty C, Stevens A, Gartlehner G, King V, Kamel C (2016). Cochrane rapid reviews methods group to play a leading role in guiding the production of informed high-quality, timely research evidence syntheses. Syst Rev..

[7] Haby MM, Chapman E, Clark R, Barreto J, Reveiz L, Lavis JN (2016). What are the best methodologies for rapid reviews of the research evidence for evidence-informed decision making in health policy and practice: a rapid review. Health Res Policy Syst.

[8] Kaltenthaler E, Cooper K, Pandor A, Martyn-St James M, Chatters R, Wong R (2016). The use of rapid review methods in health technology assessments: 3 case studies. BMC Med Res Methodol.

[9] Patnode CD, Eder ML, Walsh ES, Viswanathan M, Lin JS (2018). The use of rapid review methods for the U.S. preventive services task force. Am J Prev Med.

[10] Shemilt I, Khan N, Park S, Thomas J (2016). Use of cost-effectiveness analysis to compare the efficiency of study identification methods in systematic reviews. Syst Rev..

[11] Pham MT, Waddell L, Rajic A, Sargeant JM, Papadopoulos A, McEwen SA (2016). Implications of applying methodological shortcuts to expedite systematic reviews: three case studies using systematic reviews from Agri-food public health. Res Syn Meth..

[12] Eden J, Levit L, Berg A, Morton S, editors. Finding what works in health care: standards for systematic reviews. Washington, DC: National Academies Press; 2011. 10.17226/13059. 24983062

[13] Doust JA, Pietrzak E, Sanders S, Glasziou PP (2005). Identifying studies for systematic reviews of diagnostic tests was difficult due to the poor sensitivity and precision of methodologic filters and the lack of information in the abstract. J Clin Epidemiol.

[14] Wilhelm BJ, Rajic A, Greig J, Waddell L, Trottier G, Houde A (2011). A systematic review/meta-analysis of primary research investigating swine, pork or pork products as a source of zoonotic hepatitis E virus. Epidemiol Infect.

[15] Mathes T, Klassen P, Pieper D (2017). Frequency of data extraction errors and methods to increase data extraction quality: a methodological review. BMC Med Res Methodol.

[16] Dorée C, Hausner E, Mathisen M, Waffenschmidt S. Value of using different search approaches. http://vortal.htai.org/?q=node/993. Accessed 16.03.2016.

[17] Köhler M, Haag S, Biester K, Brockhaus AC, McGauran N, Grouven U (2015). Information on new drugs at market entry: retrospective analysis of health technology assessment reports versus regulatory reports, journal publications, and registry reports. BMJ..

[18] Waffenschmidt S, Hausner E, Sieben W, Jaschinski T, Knelangen M, Overesch I (2018). Effective study selection using text mining or a single-screening approach: a study protocol. Syst Rev.

