# Supplementary material for: Single screening versus conventional double screening for study selection in systematic reviews: a methodological systematic review
Source: BMC Med Res Methodol. 2019 Jun 28;19:132. doi: 10.1186/s12874-019-0782-0 (PMC6599339; doi:10.1186/s12874-019-0782-0)
Supplement: Supplementary file 1 — Appendix A: Search strategy (DOCX 22 kb) [file 12874_2019_782_MOESM1_ESM.docx]

**Appendix A – Search strategy**

**Medline (OVID)**

| OVID Medline Epub Ahead of Print, In-Process & Other Non-Indexed Citations, Ovid MEDLINE(R) Daily and Ovid MEDLINE(R) 1946 to Present   \| **#** \| **Searches** \| **Results** \| \| --- \| --- \| --- \| \| 1 \| systematic review*.ti,ab. \| 129698 \| \| 2 \| Review Literature as Topic/ \| 7554 \| \| 3 \| 1 or 2 \| 133849 \| \| 4 \| ((identifi* or identify* or identification or relevant screening or filtering) adj3 (study or trials or studies or articles or abstract or citation or literature)).ti,ab. \| 216152 \| \| 5 \| (eligibility or omission* or omitted or ascertainment or missed or discordancy or disagreed or workload or false negative or accuracy or rejection).ti,ab. \| 553090 \| \| 6 \| 3 and 4 and 5 \| 3265 \| \| 7 \| ((identifi* or identify* or identification or relevant screening or filtering) adj2 (study or trials or studies or articles or abstract or citation or literature)).ti,ab. \| 126134 \| \| 8 \| 3 and 5 and 7 \| 2723 \| \| 9 \| systematic review*.ti,ab. \| 129698 \| \| 10 \| Review Literature as Topic/ \| 7554 \| \| 11 \| 9 or 10 \| 133849 \| \| 12 \| (identifi* of trials or identify* studies or study identification or relevant studies).ti,ab. \| 17151 \| \| 13 \| (screening of articles or screening articles or abstract screening or citation screening or literature filtering).ti,ab. \| 451 \| \| 14 \| 12 or 13 \| 17567 \| \| 15 \| (eligibility or omission* or omitted or ascertainment or missed or discordancy or disagreed or workload).ti,ab. \| 118669 \| \| 16 \| (false negative or accuracy or rejection).ti,ab. \| 441418 \| \| 17 \| 15 or 16 \| 553090 \| \| 18 \| 11 and 14 and 17 \| 991 \| |
| --- | --- | --- | --- | --- | --- | --- | --- | --- | --- | --- | --- | --- | --- | --- | --- | --- | --- | --- | --- | --- | --- | --- | --- | --- | --- | --- | --- | --- | --- | --- | --- | --- | --- | --- | --- | --- | --- | --- | --- | --- | --- | --- | --- | --- | --- | --- | --- | --- | --- | --- | --- | --- | --- | --- | --- | --- | --- |

**PubMed (NLM)**

| Search | Query | Items found |
| --- | --- | --- |
| [#2](https://www.ncbi.nlm.nih.gov/pubmed) | Search 15845330[pmid] | [1](https://www.ncbi.nlm.nih.gov/pubmed/?cmd=HistorySearch&querykey=2) |
| [#3](https://www.ncbi.nlm.nih.gov/pubmed) | Similar articles for PubMed (Select 15845330) | [106](https://www.ncbi.nlm.nih.gov/pubmed/?cmd=HistorySearch&querykey=3) |
| [#4](https://www.ncbi.nlm.nih.gov/pubmed) | Select 20 document(s) | [20](https://www.ncbi.nlm.nih.gov/pubmed/?cmd=HistorySearch&querykey=4) |
| [#5](https://www.ncbi.nlm.nih.gov/pubmed) | Search 12111924[pmid] | [1](https://www.ncbi.nlm.nih.gov/pubmed/?cmd=HistorySearch&querykey=5) |
| [#6](https://www.ncbi.nlm.nih.gov/pubmed) | Similar articles for PubMed (Select 12111924) | [197](https://www.ncbi.nlm.nih.gov/pubmed/?cmd=HistorySearch&querykey=6) |
| [#7](https://www.ncbi.nlm.nih.gov/pubmed) | Select 20 document(s) | [20](https://www.ncbi.nlm.nih.gov/pubmed/?cmd=HistorySearch&querykey=7) |
| [#8](https://www.ncbi.nlm.nih.gov/pubmed) | Search 27285733[pmid] | [1](https://www.ncbi.nlm.nih.gov/pubmed/?cmd=HistorySearch&querykey=8) |
| [#9](https://www.ncbi.nlm.nih.gov/pubmed) | Similar articles for PubMed (Select 27285733) | [105](https://www.ncbi.nlm.nih.gov/pubmed/?cmd=HistorySearch&querykey=9) |
| [#10](https://www.ncbi.nlm.nih.gov/pubmed) | Select 20 document(s) | [20](https://www.ncbi.nlm.nih.gov/pubmed/?cmd=HistorySearch&querykey=10) |
| [#11](https://www.ncbi.nlm.nih.gov/pubmed) | Search 26911333[pmid] | [1](https://www.ncbi.nlm.nih.gov/pubmed/?cmd=HistorySearch&querykey=11) |
| [#12](https://www.ncbi.nlm.nih.gov/pubmed) | Similar articles for PubMed (Select 26911333) | [106](https://www.ncbi.nlm.nih.gov/pubmed/?cmd=HistorySearch&querykey=12) |
| [#13](https://www.ncbi.nlm.nih.gov/pubmed) | Select 20 document(s) | [20](https://www.ncbi.nlm.nih.gov/pubmed/?cmd=HistorySearch&querykey=13) |
| [#14](https://www.ncbi.nlm.nih.gov/pubmed) | Search 27535658[pmid] | [1](https://www.ncbi.nlm.nih.gov/pubmed/?cmd=HistorySearch&querykey=14) |
| [#15](https://www.ncbi.nlm.nih.gov/pubmed) | Similar articles for PubMed (Select 27535658) | [106](https://www.ncbi.nlm.nih.gov/pubmed/?cmd=HistorySearch&querykey=15) |
| [#16](https://www.ncbi.nlm.nih.gov/pubmed) | Select 20 document(s) | [20](https://www.ncbi.nlm.nih.gov/pubmed/?cmd=HistorySearch&querykey=16) |
| [#17](https://www.ncbi.nlm.nih.gov/pubmed) | Search #4 or #7 or #10 or #13 or #16 | [90](https://www.ncbi.nlm.nih.gov/pubmed/?cmd=HistorySearch&querykey=17) |
