# Supplementary material for: Single screening versus conventional double screening for study selection in systematic reviews: a methodological systematic review
Source: BMC Med Res Methodol. 2019 Jun 28;19:132. doi: 10.1186/s12874-019-0782-0 (PMC6599339; doi:10.1186/s12874-019-0782-0)
Supplement: Supplementary file 2 — Appendix B: Detailed eligibility criteria (DOCX 14 kb) [file 12874_2019_782_MOESM2_ESM.docx]

**Appendix B - Eligibility criteria**

| Population/Setting | Any screening step |
| --- | --- |
| Intervention | Single screening |
| Comparison | Double screening |
| Outcome | At least one quantitative measure for missing studies |
| Study types included in the screenings | No restrictions |
| Excluded | - Evaluations involving students or persons without screening experience - Text-mining tools - Languages other than German and English |
