# Supplementary material for: Single screening versus conventional double screening for study selection in systematic reviews: a methodological systematic review
Source: BMC Med Res Methodol. 2019 Jun 28;19:132. doi: 10.1186/s12874-019-0782-0 (PMC6599339; doi:10.1186/s12874-019-0782-0)
Supplement: Supplementary file 4 — Appendix D: List of excluded references (full text) sorted by reasons (DOCX 21 kb) [file 12874_2019_782_MOESM4_ESM.docx]

**Appendix D – List of excluded references (full text) sorted by reasons**

Quantified measures were either not reported or could not be calculated

1. Downs SH, Parry JE, Upton PA, Broughan JM, Goodchild AV, Nunez-Garcia J, et al. Methodology and preliminary results of a systematic literature review of ante-mortem and post-mortem diagnostic tests for bovine tuberculosis. Prev Vet Med. 2018;153:117-26.

2. Rosen L, Suhami R. The art and science of study identification: a comparative analysis of two systematic reviews. BMC Med Res Methodol. 2016;16:24.

3. Rathbone J, Hoffmann T, Glasziou P. Faster title and abstract screening? Evaluating Abstrackr, a semi-automated online screening program for systematic reviewers. Syst. 2015;4:80.

4. Rathbone J, Albarqouni L, Bakhit M, Beller E, Byambasuren O, Hoffmann T, et al. Expediting citation screening using PICo-based title-only screening for identifying studies in scoping searches and rapid reviews. Syst. 2017;6:233.

5. Ouzzani M, Hammady H, Fedorowicz Z, Elmagarmid A. Rayyan-a web and mobile app for systematic reviews. Syst. 2016;5:210.

6. Olofsson H, Brolund A, Hellberg C, Silverstein R, Stenstrom K, Osterberg M, et al. Can abstract screening workload be reduced using text mining? User experiences of the tool Rayyan. Res. 2017;8:275-80.

7. Norgaard MF, Grauslund J. Automated Screening for Diabetic Retinopathy - A Systematic Review. Ophthalmic Res. 2018;16:16.

8. Mo Y, Kontonatsios G, Ananiadou S. Supporting systematic reviews using LDA-based document representations. Syst. 2015;4:172.

9. Mateen FJ, Oh J, Tergas AI, Bhayani NH, Kamdar BB. Titles versus titles and abstracts for initial screening of articles for systematic reviews. Clin Epidemiol. 2013;5:89-95.

10. Gates A, Johnson C, Hartling L. Technology-assisted title and abstract screening for systematic reviews: a retrospective evaluation of the Abstrackr machine learning tool. Syst. 2018;7:45.

11. Ford AC, Guyatt GH, Talley NJ, Moayyedi P. Errors in the conduct of systematic reviews of pharmacological interventions for irritable bowel syndrome. Am J Gastroenterol. 2010;105:280-8.

The publication was not an original evaluation

1. O'Mara-Eves A, Thomas J, McNaught J, Miwa M, Ananiadou S. Using text mining for study identification in systematic reviews: a systematic review of current approaches. Syst. 2015;4:5.

2. Lefebvre C, Glanville J, Wieland LS, Coles B, Weightman AL. Methodological developments in searching for studies for systematic reviews: past, present and future? Systematic reviews. 2013;2:78.

3. Cooper C, Booth A, Britten N, Garside R. A comparison of results of empirical studies of supplementary search techniques and recommendations in review methodology handbooks: a methodological review. Syst. 2017;6:234.

The evaluations involved students or persons without screening experience

1. Ng L, Pitt V, Huckvale K, Clavisi O, Turner T, Gruen R, et al. Title and Abstract Screening and Evaluation in Systematic Reviews (TASER): a pilot randomised controlled trial of title and abstract screening by medical students. Syst. 2014;3:121.

2. Nama N, Iliriani K, Xia MY, Chen BP, Zhou LL, Pojsupap S, et al. A pilot validation study of crowdsourcing systematic reviews: update of a searchable database of pediatric clinical trials of high-dose vitamin D. Transl. 2017;6:18-26.

3. Mortensen ML, Adam GP, Trikalinos TA, Kraska T, Wallace BC. An exploration of crowdsourcing citation screening for systematic reviews. Res. 2017;8:366-86.

4. Cooper M, Ungar W, Zlotkin S. An assessment of inter-rater agreement of the literature filtering process in the development of evidence-based dietary guidelines. Public Health Nutr. 2006;9:494-500.
